# Evaluating implementation strategies for essential newborn care interventions in low- and low middle-income countries: a systematic review

**DOI:** 10.1093/heapol/czaa122

**Published:** 2020-11-06

**Authors:** Kimberly Peven, Debra Bick, Edward Purssell, Torill Alise Rotevatn, Jane Hyldgaard Nielsen, Cath Taylor

**Affiliations:** 1 Florence Nightingale Faculty of Nursing, Midwifery & Palliative Care, King’s College London, 57 Waterloo Road, London SE1 8WA, UK; 2 Warwick Clinical Trials Unit, University of Warwick, UK; 3 School of Health Sciences, City, University of London, London, UK; 4 Public Health and Epidemiology Group, Department of Health Science and Technology, Aalborg University, Aalborg, Denmark; 5 Department of Midwifery, University College of Northern Denmark, Aalborg, Denmark; 6 School of Health Sciences, University of Surrey, Guildford, UK

**Keywords:** Child health, developing countries, health systems research, health workers, infant mortality, international health, maternal and child health, strategy, systematic reviews

## Abstract

Neonatal mortality remains a significant health problem in low-income settings. Low-cost essential newborn care (ENC) interventions with proven efficacy and cost-effectiveness exist but have not reached high coverage (≥90%). Little is known about the strategies used to implement these interventions or how they relate to improved coverage. We conducted a systematic review of implementation strategies and implementation outcomes for ENC in low- and low middle-income countries capturing evidence from five medical and global health databases from 1990 to 2018. We included studies of implementation of delayed cord clamping, immediate drying, skin-to-skin contact (SSC) and/or early initiation of breastfeeding implemented in the first hour (facility-based studies) or the 1st day (community-based studies) of life. Implementation strategies and outcomes were categorized according to published frameworks: Expert Recommendations for Implementing Change and Outcomes for Implementation Research. The relationship between implementation strategies and outcomes was evaluated using standardized mean differences and correlation coefficients. Forty-three papers met inclusion criteria. Interventions included community-based care/health promotion and facility-based support and health care provider training. Included studies used 3–31 implementation strategies, though the consistency with which strategies were applied was variable. Conduct educational meetings was the most frequently used strategy. Included studies reported 1–4 implementation outcomes with coverage reported most frequently. Heterogeneity was high and no statistically significant association was found between the number of implementation strategies used and coverage of ENC. This review highlights several challenges in learning from implementation of ENC in low- and low middle-income countries, particularly poor description of interventions and implementation outcomes. We recommend use of UK Medical Research Council guidelines (2015) for process evaluations and checklists for reporting implementation studies. Improved reporting of implementation research in this setting is necessary to learn how to improve service delivery and outcomes and thereby reduce neonatal mortality.

**KEY MESSAGES**

- This is the first systematic review to examine implementation strategies and outcomes for essential newborn care interventions in lower income countries, finding poor reporting of implementation strategies and outcomes.
- Implementation efforts to integrate essential newborn care interventions in low- and low middle-income countries have used a wide variety of implementation strategies, however, the detail with which the strategies are reported is insufficient for replication or learning.
- Implementation outcomes reported in the literature are limited—mostly focusing on coverage and omitting acceptability and other quality measures—restricting the ability to learn from previous implementation efforts.
- There is an urgent need to improve reporting of implementation research in this setting to learn how to improve service delivery and outcomes and thereby reduce neonatal mortality.

## Introduction

Globally, in 2018, 2.5 million babies died in the 1st month of life, with most of these deaths occurring in the least developed countries and about a third occurring on the day of birth (UNICEF *et al.*, 2019). Improved care around the time of birth, including essential newborn care (ENC) as prioritized by the World Health Organisation (WHO, 2017), could potentially prevent many of these deaths. In 2005, the Lancet Neonatal Survival Series ‘Call to Action’ called for high coverage of interventions to reduce neonatal mortality (Martines *et al.*, 2005). Over a decade later, however, coverage of effective newborn health interventions remains low overall (Bhutta *et al.*, 2014). Proctor *et al.* (2013) define coverage (or penetration) as the integration of a practice or in the case of ENC interventions: the number of babies who received the intervention out of all live births.

An evaluation of global performance in newborn health by Darmstadt *et al.* (2014) in 2014 found minimal progress in implementation and evaluation since the 2005 call to action. Evidence on effective methods for integration of newborn care into health systems in the low-income country context is lacking, limiting opportunities for learning—as we only know if something works and not why, how or for whom (Darmstadt *et al.*, 2014). Well-established evidence of intervention efficacy has not translated to high coverage in low- and middle-income countries (LMICs) (The World Bank, 2019). This knowledge-to-practice gap is consistent with findings across other public health domains, where translation of research evidence to practice is slow and haphazard, and has cost lives (Eccles *et al.*, 2009). The World Health Organization (WHO, 2013) has identified evaluating the effectiveness of different strategies to implement postnatal care recommendations as a high-priority research gap. Furthermore, a recent publication has called for an increase in implementation research in global health to improve health outcomes and bridge the gap among research, policy and practice (Theobald *et al.*, 2018).

Although implementation research has recently been prioritized by policy makers, implementers and researchers (Ghaffar *et al.*, 2017), including the launch of an implementation research platform within WHO (2016), inconsistent terms and definitions of implementation strategies have complicated the field (McKibbon *et al.*, 2010). To improve conceptual clarity and allow for improved implementation research and reproducibility, Powell *et al.* (2015) described 73 implementation strategies compiled by a panel of experts in implementation science and clinical practice (health and mental health). The panel rated the relative importance and feasibility of each strategy and clustered them into nine distinct groups using hierarchical cluster analysis (Waltz *et al.*, 2015). This provides a framework for assessing implementation strategies used in deploying ENC interventions in low- and low middle-income countries.

Furthermore, implementation effectiveness must be measured distinctly from clinical effectiveness to increase our understanding of intervention performance in different contexts. As implementation success depends on local factors (Damschroder *et al.*, 2009), understanding implementation outcomes is necessary to distinguish ineffective interventions from poor deployment of interventions. As such, recording and reporting implementation outcomes is an important addition to recording and reporting morbidity and mortality outcomes as we translate and test interventions with proven efficacy across settings (Proctor *et al.*, 2013). However, poor descriptions of implementation and lack of reporting important outcomes is a recognized problem and contributor to research waste (Glasziou *et al.*, 2014).

There is currently no acknowledged ‘gold standard’ approach to support implementation and sustainability of ENC interventions in low- and low middle-income country settings. To synthesize understanding, we present results from a systematic review of the literature on implementation strategies and implementation outcomes for deploying ENC interventions in low- and low middle-income countries. Specifically, our objectives were to:

1. identify and describe which implementation strategies and outcomes are reported for implementing ENC interventions in low- and low middle-income countries,
2. determine the relationship between implementation strategies and coverage of ENC interventions in low- and low middle-income countries.

## Methods

**Figure 1:**
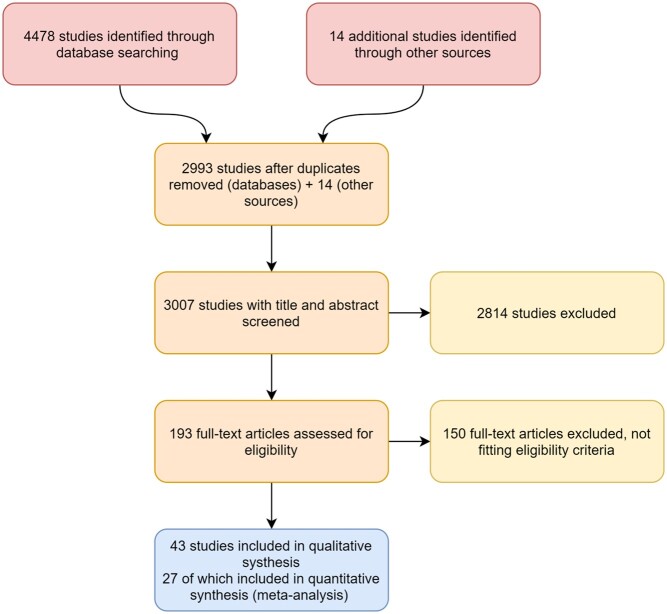
PRISMA flow diagram (Moher *et al.*, 2009)

We performed a systematic review of the literature to identify studies reporting on the implementation of ENC interventions for healthy newborns in low- and low middle-income countries (The World Bank, 2019). We have reported results of the review according to Preferred Reporting Items for Systematic Reviews and Meta-analyses (PRISMA) guidelines with the PRISMA flow diagram presented in Figure 1 and the PRISMA checklist in the Supplementary file.

### Search methods

Systematic searches were carried out for literature published from January 1990 to 22 June 2018 in the health and social care databases: MEDLINE, EMBASE, CINAHL and Cochrane Central databases as well as the Global Health Library. The search strategy (shown in full in the Supplementary file) incorporated key terms related to ENC (e.g. breastfeeding, drying, SSC and delayed cord clamping), implementation outcomes (e.g. acceptability, adoption, appropriateness), countries (low- and low middle-income countries) and newborns (e.g. newborn, neonate).

### Study selection

#### Population

All studies of interventions targeting healthy newborns in the first hour (for facility-based interventions) or day (for community-based interventions) of life in low- and low middle-income countries were included. The population was restricted to newborns not requiring special care so the interventions would be widely applicable to most or all settings without requiring highly skilled workers, advanced treatments or significant infrastructure. Newborns not requiring special care were defined as per the WHO Safe Birth Checklist: born not more than 1 month prematurely, with birth weight >2500 g, who did not need antibiotics or require resuscitation at birth (Spector *et al.*, 2012). Studies which only targeted newborns who required special care were excluded as care needs are likely to be different. Studies which targeted all newborns (thereby including some newborns requiring special care and monitoring) were included.

#### Intervention

Studies examining the implementation of ENC interventions (immediate and thorough drying, immediate SSC, delayed cord clamping and early initiation of breastfeeding) were eligible if they were implemented within the first hour of life for facility-based interventions, and the 1st day of life for community-based interventions, as neonatal mortality is highest in this time frame and most recommended ENC interventions are intended to be implemented immediately after birth (Salam *et al.*, 2014). Interventions at the community level, facility level or both levels were included. These interventions are recommended for all births (community and facility) (World Health Organization, 2017), can be implemented without advanced infrastructure, and are accepted as effective and cost-effective (Darmstadt *et al.*, 2005).

#### Types of studies

The review included peer-reviewed, empirical quantitative and qualitative study designs conducted in low- and low middle-income countries which described the implementation of a relevant intervention. No restrictions were placed on study sample size or language.

To remain relevant for implementation of current evidence-based interventions in contemporary health systems, inclusion was restricted to studies published from 1990 to 2018.

#### Selection criteria

##### Inclusion criteria

- Peer-reviewed, primary research
- Studies conducted in low- and low middle-income countries [defined by The World Bank (2019) at the time of the systematic review]
- Studies which included ENC interventions (immediate and thorough drying, immediate SSC, delayed cord clamping and early initiation of breastfeeding) in the first hour of life (facility-based interventions) or the 1st day (24 h) of life (community-based interventions)
- Studies which included an intervention provided directly to the mother–newborn dyad OR measured outcomes in the mother–newborn dyad
- Studies which included an implementation outcome [defined by Proctor *et al.* (2011)].

##### Exclusion criteria

- Studies which focused entirely on premature, low birth weight, or at-risk newborns or newborns requiring special care and monitoring
- Studies which focused entirely on caesarean section deliveries, adolescent mothers or mothers with special needs (e.g. HIV, high risk pregnancies)

#### Selection process.

Papers resulting from the search were first screened by reviewing the title and abstract and then by reviewing the full text (*KP*). Fifty per cent of papers were randomly selected, using the R package metagear (Lajeunesse, 2017), to be screened independently by a second reviewer at each stage (EP, CT). Disagreements between reviewers were resolved through discussion or involvement of a third study team member.

Data from each paper were extracted independently by two reviewers using a standardized proforma including the name and a short description of the intervention, study design, implementer, setting, population, key findings, implementation strategies and implementation outcomes (KP, TAR, JHN). All references of included studies were reviewed and further information on implementation strategies applied was sought from additional programme documents and protocols where identified. Quality appraisals were conducted independently by two reviewers for each paper (KP, TAR, JHN) using the Joanna Briggs Institute Critical Appraisal Tools (Aromataris and Munn, 2017). No papers were excluded on the basis of the quality appraisal. Inconsistencies between reviewers were resolved through discussion and consensus or involvement of a third team member.

After reviewing the literature for implementation strategy frameworks, particularly those with relevance to maternal and newborn health or the low-income country setting, we chose a framework by the Expert Recommendations for Implementing Change project (Powell *et al.*, 2015). This framework was the most comprehensive we identified (including 73 strategies) and was developed systematically with input from clinical and implementation science stakeholders. Strategies are mapped to nine clusters encompassing strategies ranging from clinician reminders and use of data experts to using mass media or changing record systems (Waltz *et al.*, 2015). During data extraction, we compared strategies used in implementing interventions in included papers to the definitions of implementation strategies described by Powell *et al.* (2015). A table of the 73 strategies and their definitions is included in a Supplementary file.

### Analysis

A narrative descriptive approach was taken to summarize interventions and describe and synthesize implementation strategies. We report the number of implementation outcomes [defined by Proctor *et al.* (2011)] reported by each study and describe the frequency with which implementation outcomes are reported. As there was some similarity of interventions within the implementation setting (community, facility or mixed), interventions are described according to their setting. Examples of implementation strategy application are described for the most frequently used strategy within each cluster designated by Waltz *et al.* (2015).

#### Quantitative analysis

The implementation strategies applied within included papers were categorised according to the typology provided by Powell *et al.*, (2015). Waltz *et al.* (2015) provide an importance rating and a feasibility rating for each strategy. Ratings are on a scale of one (relatively unimportant/not at all feasible) to five (extremely important/extremely feasible). We assigned a mean importance rating (average of the importance ratings for each strategy used in the paper) to each paper. Furthermore, each paper was assessed for reporting implementation outcomes as defined by Proctor *et al.* (2013) (Supplementary file).

Papers reporting two coverage outcomes (e.g. either before and after or in a control and intervention) were included in the quantitative analysis. The term coverage is used to describe the domain in line with maternal, newborn and child health literature. Proctor *et al.* (2011) previously labelled this domain as ‘penetration’ which they defined as “integration of a practice within a service setting and its sub-systems” (p. 70). Though Proctor *et al.* (2011) found the term ‘penetration’ to be infrequently used in the implementation literature, the construct was often/usually addressed using other terms.

To address objective two and examine the relationship between implementation strategies and coverage of ENC, we first estimated the magnitude of each intervention’s effects on coverage of ENC practices by calculating effect sizes (standardized mean differences) for each coverage outcome using Cohen’s *d*. We calculated Pearson’s correlation coefficients to examine the relationships between the total number of strategies used in implementation and the coverage effect size as well as the mean importance rating of the strategies used and the coverage effect size. In addition, we calculated correlation coefficients for the per cent of strategies used within each cluster [as defined by Waltz *et al.* (2015)] and the coverage effect size. All quantitative analyses were carried out in R Statistical Software (R Core Team, 2018).

To detect possible publication bias, funnel plots of fitted meta-analytic models with standard error, sampling variance, inverse standard error and inverse sampling variance as predictors were visually examined. A random-effects meta-regression model with the standard error as the predictor revealed no significant asymmetry (*z* = 0.15, *P* = 0.89).

## Results

### Included studies

Of the 3007 unique citations identified in the search, 2814 were excluded after screening the title and abstract. The remaining 193 were assessed by reviewing the full text. Inclusion criteria were not met for 150 citations, and 43 were thus included in the narrative synthesis and 27 of these were additionally included in the quantitative analysis (Figure 1). While the search strategy identified several non-English publications, including papers written in Portuguese, Spanish and French, no non-English papers met all inclusion criteria. Characteristics of included studies are summarized in Table 1.

**Table 1:** Characteristics of included studies

First author and year	ENC component	Study design, methods, sample size (for studies included in quantitative analysis)	Intervention	Country and setting	Implementing body; time between start of implementation and final follow-up
Arabi et al. (2018)	Immediate drying	Analytic cross-sectional Interviews and report review Baseline *n*=1305 Endline *n*=3040	Helping Babies Breathe (HBB) training: Training in neonatal resuscitation and postnatal care and regular peer to peer skills practice	Sudan Rural, village midwives	Ministry of Health and Maternity Hospital in Ireland 18 months
Aryeetey and Antwi (2013)	Breastfeeding	Analytic cross-sectional Interviews Pregnant women *n* = 60 Clinical staff *n* = 90 Recently delivered women *n* = 150	BFHI: a global hospital-based initiative to implement practices that protect, promote and support breastfeeding	Ghana Public-managed facilities in the urban Accra Metropolis	Central government 7 years
Baqui et al. (2008)	Breastfeeding	Cluster RCT Surveys Home care arm *n* = 14 769 Community care arm *n*=16 325 Control arm *n*=15 350	Project for advancing the health of newborns and mothers (Projahnmo): Community health worker home-visits for antenatal and postnatal care, care seeking promoted through group sessions by community mobilisers	Bangladesh Community-based project in rural sub districts of Sylhet, Bangladesh	University with central and local government and other partners 2.5 years
Bhutta et al. (2008)	Breastfeeding	Quasi-experimental Surveys Intervention *n* = 395 Control *n* = 375	Lady Health Workers (LHW) and Dais (Traditional birth attendants): Pilot of training for home-based basic newborn care, community organization and mobilization and group education	Pakistan Community-based intervention in rural Hala and Matiari sub districts	University with central and local government 2 years
Bhutta et al. (2011)	Breastfeeding	Cluster RCT Surveys Intervention *n*=12 517 Control *n* = 11 568	LHWs and Dais (Traditional birth attendants): Training for home-based basic newborn care, community organization and mobilization and group education	Pakistan Community-based intervention in rural Hala and Matiari sub districts	University with central and local government 2 years
Callaghan-Koru et al. (2013)	Breastfeeding Skin-to-skin	Quasi-experimental Surveys Baseline *n* = 903 Final follow-up *n* = 900	Community-based maternal and newborn care program (CBMNC): A package of facility and community-based interventions, health surveillance assistants (HAS) trained to make pregnancy and postnatal visits, facility worker training	Malawi Three rural districts: Thyolo, Dowa, Chitipa	Ministry of health with support from Save the Children's Saving Newborn Lives and UNICEF <3 years
Callaghan-Koru et al. (2016)	Drying Skin-to-skin	Quasi-experimental Surveys Baseline *n* = 218 Final follow-up *n* = 214	A multilevel intervention to promote early SSC and exclusive breastfeeding: Health workers in facilities and surrounding communities were trained to promote early SSC and exclusive breastfeeding for all babies born at home or in facilities	Ethiopia 10 health centres and their catchment areas in the Tigray, Oromia, Amhara and SNNP	Central government with support from Maternal and Child Health Integrated Program (MCHIP) <2 years
Darmstadt et al. (2006)	Skin-to-skin	Qualitative In-depth interviews, focus groups	Community-based skin to skin care: Community health workers delivered a package of socioculturally contextualized behaviour change interventions through community meetings and household visits	India Community-based intervention in Shivgarh, a rural block of Uttar Pradesh	Health facility with University partner +1 year
Darmstadt et al. (2010)	Breastfeeding Drying	Cluster RCT Surveys Intervention *n* = 9987 Control *n* = 11 153	Projahnmo-2: Trained CHWs perform ANC and PNC home visits and manage neonatal illness by clinical algorithm	Bangladesh Community-based project in Mirzapur, Bangladesh	Health facility, research centre and university 2 years
Delaney et al. (2017)	Breastfeeding Skin-to-skin	Analytic cross-sectional Observations	BetterBirth trial: An intervention for the sustainable adoption of the SCC through peer coaching	India Facilities in Uttar Pradesh	US Health System Innovation Center 8 months
Dasgupta et al. (1997)	Breastfeeding	Quasi-experimental Observations Baseline *n* = 102 Final follow-up *n* = 102	BFHI: a global hospital-based initiative to implement practices that protect, promote and support breastfeeding	India Urban health facility	Health facility 6 months
Ekirapa-Kiracho et al. (2017)	Breastfeeding Drying Skin-to-skin	Economic evaluation Incremental cost analysis from programme records	Uganda Newborn Study (UNEST): Implementation of a community health worker maternal child health home visit package and facility strengthening	Uganda Eastern Uganda, rural and peri-urban areas on Iganga and Mayuge districts	Central and local government with other partners 2 years
Fathima et al. (2015)	Breastfeeding	Analytic cross-sectional Surveys ASHAs *n* = 300 Women *n* = 1200	ASHA: Community health volunteer scheme, women selected by communities facilitate access to facilities, provide community health services and mobilize communities for change	India Community-based intervention in Karnataka State	Central government +5 years
Goudar et al. (2012)	Breastfeeding Skin-to-skin	Cluster RCT Data form review Baseline *n* = 5912 Endline *n* = 6163	ENC training: Training for auxiliary nurse midwives, traditional birth attendants and other community birth attendants were trained in clean delivery practices, neonatal resuscitation and ENC	India Rural, community-level	Global research network and government officials 3 years
Greco et al. (2017)	Breastfeeding Skin-to-skin	Economic evaluation Incremental cost analysis from programme records	CBMNC: A package of facility and community-based interventions, health surveillance assistants (HAS) trained to make pregnancy and postnatal visits, facility worker training	Malawi Three rural districts: Thyolo, Dowa, Chitipa	Ministry of health with support from Save the Children's Saving Newborn Lives and UNICEF <3 years
Hirschhorn et al. (2015)	Breastfeeding	Quasi-experimental Observations Phase 1 Before *n* = 23 Phase 1 After *n* = 23 Phase 2 Before *n* = 522 Phase 2 After *n* = 409	BetterBirth: A 3-day staff training and introduction of WHO SCC	India Health facilities in Uttar Pradesh	Health facilities with Population Services International 1 month
Hirschhorn et al. (2018)	Breastfeeding Skin-to-skin	Mixed-methods/qualitative Observation Tool to Inform Support (OTIS)	WHO SCC: Opportunity–Ability–Motivation plus Supplies framework was integrated into coaching to improve delivery of essential birth practices	India Facilities in Uttar Pradesh	US Health System Innovation Center 8 months
Iyengar et al. (2014)	Breastfeeding Drying	Quasi-experimental Observations Facilities *n* = 44	Parijaat: A collaborative for improving the quality of facility deliveries in high-volume facilities through training, quality monitoring and clinician reminders	India Public facilities in Rajasthan	State Government with Action Research & Training for Health and United Nations Population Fund <2 years
Jennings et al. (2015)	Breastfeeding Skin-to-skin	Cluster RCT Baseline Control *n* = 56 Baseline Intervention *n* = 95 Final follow-up Control *n* = 99 Final follow-up Intervention *n* = 161	Job Aids to Improve Facility-based Postnatal Counselling and Care: Pictorial Job Aids were developed for Facility staff to use in Postnatal counselling, training and supportive supervision was provided to Improve communication and counselling skills	Benin Rural health facilities in Zou and Collines region	Central government with USAID <1 month
Kamath-Rayne et al. (2017)	Breastfeeding Skin-to-skin Cord clamping	Analytic cross-sectional Observations Deliveries attended by individuals without HBB training *n* = 156 Deliveries attended by HBB trainees *n* = 94	HBB training: Training in neonatal resuscitation and ENC	Honduras Rural community hospital	US-based HBB master trainers 5 months
Karim et al. (2013)	Breastfeeding Drying Skin-to-skin	Quasi-experimental Surveys Baseline *n* = 1,404 Final follow-up *n* = 1404	Health Extension Program (HEP): A community health worker programme to provide universal primary health care access including postnatal visits	Ethiopia Community level intervention studied in Tigray, Amhara, Oromia, SNNPR	Central government 2 years
Kayemba et al. (2012)	Breastfeeding	Qualitative Survey, in-depth interview, focus groups, document review VHT members (survey) *n* = 436	Integrated Community Case Management (iCCM): A 6 days of training for village health team volunteers to care for babies 0–59 days in the community	Uganda Kiboga, Kyankwanzi and Hoima districts	Central government +5 months
Kumar et al. (2008)	Breastfeeding Drying Skin-to-skin	Cluster RCT Surveys ENC arm *n*=1581 ENC+Thermospot arm II *n* = 1135 Control arm *n* = 1143	Community-based behaviour change management: Community health workers delivered a package of socioculturally contextualized behaviour change interventions through community meetings and household visits	India Community-based intervention in Shivgarh, a rural block of Uttar Pradesh	Health facility with University partner +1 year
Kung’u et al. (2018)	Breastfeeding Cord clamping	Quasi-experimental Surveys Baseline *n* = 2905 Endline *n* = 2570	Community-based maternal and neonatal health and nutrition project A set of demonstration projects in four countries, based on need, context and policies, to demonstrate how proven nutrition interventions could be integrated into health programs to improve practices during pregnancy, birth and postpartum	Ethiopia, Kenya, Senegal Community and facility levels	NGO, government and other partners 2 years
Lefevre et al. (2013)	Breastfeeding	Economic evaluation Cost-effectiveness analysis Costs from financial records and survey of household costs Households (survey) *n* = 316	Project for advancing the health of newborns and mothers (Projahnmo): Community health worker home-visits for ANC and PNC, care seeking promoted through group sessions by community mobilisers	Bangladesh Community-based project in rural sub districts of Sylhet, Bangladesh	University with central and local government and other partners 2.5 years
Manasyan et al.(2011)	Breastfeeding Drying Skin-to-skin	Economic evaluation Incremental cost analysis from programme records	World Health Organization Essential Newborn Care Course: ENC training for facility providers	Zambia Urban facilities in the two largest cities (Lusaka, Ndola)	Central government and other partners 1 year
Myint et al. (2013)	Breastfeeding	Analytic cross-sectional Surveys and focus groups Midwives (survey) *n* = 46 Midwives (focus group) *n* = 40 Mothers (survey) *n* = 80 Mothers (focus group) *n* = 40	ENC programme: Strengthening of facility- and community-based interventions for improving maternal and neonatal health and improving case management skills of skilled birth attendants	Myanmar Facility and community-based providers in Magway Region	Central government 4 years
Nonyane et al. (2016)	Breastfeeding Drying	Quasi-experimental Surveys Baseline *n* = 625 Final follow-up *n* = 615	Community-based Newborn Care Package (CB-NCP): Training of facility and community workers, female community health volunteers make home visits to perinatal care	Nepal Community-based intervention in Bardiya district of Nepal	Central government and other partners 1.5 years
Ojofeitimi et al. (2000)	Breastfeeding	Analytic cross-sectional Surveys Women *n* = 430	BFHI: a global hospital-based initiative to implement practices that protect, promote and support breastfeeding	Nigeria Urban health facility in Ile-Ife	Health facility 5 years
Parekh et al. (2004)	Breastfeeding	Analytic cross-sectional Surveys Mothers *n* = 98	BFHI: a global hospital-based initiative to implement practices that protect, promote and support breastfeeding	India Urban hospital in Mumbai	Health facility 10 years
Patabendige and Senanayake (2015)	Breastfeeding Skin-to-skin	Analytic cross-sectional Observations of deliveries and staff questionnaire	WHO SCC: The SCC was implemented in a tertiary care hospital through training and ward visits	Sri Lanka Tertiary facility	Hospital 2 months
Potty et al. (2017)	Breastfeeding	Analytic cross-sectional Survey Round 1 *n* = 1731 Round 2 *n* = 1113 Round 3 *n* = 1159 Round 4 *n* = 1171 Round 5 *n* = 1101	Sukshema Project: Technical assistance to the National Health Mission of Karnataka to improve newborn health through staff training and mentoring and a package of tools for frontline workers to improve care and monitor implementation	India Facility and community-based interventions in rural, northern Karnataka	State government 2 years
Pradhan et al. (2011)	Breastfeeding Drying	Quasi-experimental Surveys Baseline *n* = 625 Final follow-up *n* = 615	CB-NCP: Training of facility and community workers, female community health volunteers make home visits to perinatal care	Nepal Community-based intervention in Bardiya district of Nepal	Central government and other partners 1.5 years
Prasad and Costello (1995)	Breastfeeding	Quasi-experimental Surveys Baseline *n* = 172 Early follow-up *n* = 195 Late follow-up *n*=101	Baby Friendly health education intervention: A baby friendly training intervention was implemented for staff at a district hospital	India Bihar district hospital	Health facility 6 months
Saha and Varghese (2017)	Breastfeeding	Economic analysis Incremental cost analysis Activity-based costing	Yashoda Programme: Facility-based postnatal support in high-volume facilities	India High-volume facilities studied in Rajasthan and Odisha	Government of India and Norway–India Partnership Initiative 4 years
Senarath et al. (2007)	Breastfeeding Drying Skin-to-skin	Quasi-experimental Surveys and observations Baseline survey *n* = 223 Baseline observations *n* = 24 Final follow-up survey *n* = 223 Final follow-up survey *n* = 24	Essential Newborn Care Training: A 4-day training on ENC for doctors, nurses and midwives on obstetric units at two hospitals	Sri Lanka State-sector hospitals	University 3 months
Singh et al. (2017)	Breastfeeding	Quasi-experimental Longitudinal cohort study	Enhanced Integrated Nutrition and Health Program A demonstration and replication approach for scaling up successful nutrition practices through a partnership among government systems, non-governmental organizations and community-based organizations	India Rural, community-level	NGO, Government and community-based organizations 2 years
Sinha et al. (2014)	Breastfeeding Skin-to-skin	Analytic cross-sectional Interviews and observations Mothers (interview) *n* = 320 ASHAs (interview) *n* = 61 Observations *n* = 19	Home-based Post Natal Newborn Care (HBPNC) programme by ASHAs: ASHA workers get cash incentives for six postnatal home visits for newborn care	India Community-based project in Mewat, Haryana	State government and United Nations Office for Project Services-Norway–India Partnership Initiative <2 years
Spector et al. (2012)	Breastfeeding	Quasi-experimental Observations Baseline *n* = 499 Final follow-up *n* = 795	WHO SCC Program: Education, supervision and monitoring of safe birth checklist use and engagement of local leaders	India Sub-district level birth centre in Karnataka, India	University <3 months
Spira et al. (2017)	Breastfeeding Drying Skin-to-skin	Quasi-experimental Surveys Women *n* = 4815 Clinical staff *n* = 73	Healthcare professional associations implementing essential interventions: Healthcare professional associations implemented a package of essential interventions for maternal and newborn health	Uganda Large teaching hospitals	Healthcare professional associations <6 months
Varghese et al. (2014)	Breastfeeding	Analytic cross-sectional Surveys Intervention *n* = 810 Control *n* = 842	Yashoda Programme: Facility-based postnatal support in high-volume facilities	India High-volume facilities studied in Rajasthan and Odisha	Central government and Norway-India Partnership Initiative 4 years
Waiswa et al. (2015)	Breastfeeding Drying Skin-to-skin	Cluster RCT Surveys Baseline intervention *n* = 194 Baseline control *n* = 201 Final follow-up intervention *n* = 894 Final follow-up control *n* = 893	UNEST: Implementation of a community health worker maternal child health home visit package and facility strengthening	Uganda Eastern Uganda, rural and peri-urban areas on Iganga and Mayuge districts	Central and local government with other partners 2 years
Waiswa et al. (2017)	Breastfeeding	Quasi-experimental Surveys and facility assessments First round Tanzania, intervention *n* = 101 First round Tanzania, control *n* = 106 First round Uganda, intervention *n* = 199 First round Uganda, control *n* = 281	EQUIP A systemic and collaborative quality improvement approach to increase coverage and quality of essential interventions for maternal and newborn health	Tanzania and Uganda District, facility and community levels	Ministry of Health and Department for community 2.5 years

The final 43 included papers cover 36 unique implementation efforts. Most interventions were evaluated through surveys or observations for all births in a particular time frame and location. Seven per cent (*n* = 3) were primarily qualitative studies while 12% (*n* = 5) were economic analyses and the remainder were quantitative studies (7 cluster randomized control trials, 12 cross-sectional studies and 16 quasi-experimental studies). Interventions were implemented in 18 countries: 5 countries in South Asia, 11 countries in sub-Saharan Africa, 1 in East Asia and 1 in Latin America. The time between implementation and the final follow-up reported in the study ranged from <1 month to 10 years, with a median of 2 years. The two studies specifically addressing sustainability outcomes were both reporting evaluations taking place 2 or more years after implementation. Almost all the implemented interventions included early initiation of breastfeeding (93.0%, *n* = 40), 42.0% (*n* = 18) included SSC, 30.2% (*n* = 13) included drying of the newborn, and 4.7% (*n* = 2) included delayed cord clamping. About half (*n* = 22) included only one ENC component, 18.6% (*n* = 8) studies included three components.

### Description of interventions

Thirteen interventions were implemented in the community setting, most in Asia (*n* = 8). All interventions in this category involved the training of lay or auxiliary health care workers (paid or volunteer) to conduct home visits or support home-based ENC. Two of the community-based interventions were studies of nationally implemented programmes such as the Accredited Social Health Activists (ASHA) programme in India. ASHAs are trained female community health activists working as an interface between communities and the health system (National Health Mission, 2013). As part of a strategy to reduce neonatal mortality, ASHAs provide home-based newborn care at six home visits. In the ASHA studies included in this review, Sinha *et al.* (2014) found 33% (*n* = 55) of mothers reported an ASHA visited them within 24 h of home delivery. Fathima *et al.* (2015) found 72% (*n* = 826) of women reported being visited 3 or more times by an ASHA worker in the postpartum period and that 73% (*n* = 215) of ASHAs felt effective in their ability to provide newborn advice or care.

Seventeen interventions were implemented in the facility setting, most in low middle-income countries (*n* = 15). Facility interventions mostly included training for medically qualified facility staff, implementation of checklists or job aids (e.g. pictorial counselling cards), and implementation of the Baby Friendly Hospital Initiative (BFHI). One intervention, the Yashoda Programme (Varghese *et al.*, 2014; Saha and Varghese, 2017) in India, involved lay, volunteer women to support women and newborns in high-volume facilities with at least 150 deliveries per month.

Nine of the interventions were mixed-setting: involving both community- and facility-based components. Several of the interventions involved community health worker home visits and facility improvement or training for facility staff to complement community-based activities. The Expanded Quality Management Using Information Power (EQUIP) study described by Waiswa *et al.* (2017) used joint learning sessions with community members and facility staff to allow groups to review progress and learn from each other.

### Description of implementation strategies

The number of implementation strategies used in included studies ranged from 3 to 31 with a mean of 15.8. ‘Train and educate stakeholders’ was the most frequently used cluster of implementation strategies for ENC interventions, at least 1 of the 11 strategies in the cluster was used by each included paper [definitions of each strategy are shown in the Supplementary file]. The most frequently used individual strategy, ‘conduct educational meetings’ was used in 70% (*n* = 30) of studies. Educational meetings or trainings were conducted for interventions in the community and facility settings. In community settings, educational meetings were often held for training community health workers. A community health worker intervention described by Darmstadt *et al.* (2010) included a 36-day training for community health workers on pregnancy surveillance, counselling and negotiation, ENC and management of neonatal illness. Fortnightly refresher training and monitoring were provided following the main training (Table 2).

In facility settings, educational meetings were held for facility staff with different durations and intensities. For example, Spector *et al.* (2012) describe a 1-day learning session to introduce the WHO Safe Childbirth Checklist (SCC) to hospital staff, whereas Jennings *et al.* (2015) described 3-day training for all health care personnel at intervention sites which included didactic instruction and role-play to educate personnel on content and use of counselling cards as well as interpersonal communication and quality improvement. Waiswa *et al.* (2017) implemented educational meetings at the facility and community levels, sometimes involving joint learning sessions with community members and facility staff.

While a strategy from the ‘train and educate stakeholders’ cluster was used at least once for every paper, some strategies within this cluster were not frequently used and one (‘shadow other experts’) was not used at all. The ‘provide interactive assistance’ cluster had high use across all four strategies in the cluster: facilitation (49%, *n* = 21), provide local technical assistance (35%, *n* = 15), provide clinical supervision (56%, *n* = 24) and centralize technical assistance (19%, *n* = 8). Within this cluster, the most frequently used strategy was ‘provide clinical supervision’. This strategy often integrated regular supervision into the intervention at a fixed interval (e.g. monthly supervision visits). Some papers studying a shorter clinical intervention period used clinical supervision only during an initial implementation phase. Spira *et al.* (2017) described an intervention using healthcare professional associations (e.g. the International Confederation of Midwives) to accelerate implementation of ENC. As part of the intervention, opinion leaders selected as facilitators observed clinical practice and held discussions with staff during the initial implementation phase. In contrast, in India’s Yashoda programme assessed by Varghese *et al.* (2014), supervision was an integrated part of the programme.

**Table 2:** Definition and examples of the most frequently used strategy in each cluster

Strategy	Papers^a^*N* (%)	Definition from Powell *et al.* (2015)	Examples of use from included papers
Stage implementation scale up			
8. Stage implementation scale up	22 (51%)	Phase implementation efforts by starting with small pilots or demonstration projects and gradually move to a system-wide rollout	Most papers using stage implementation scale up were pilot tests to inform future work or RCTs informed by pilot tests. For example, Bhutta *et al.* (2008) described a pilot study which informed the RCT conducted by Bhutta *et al.* (2011). Both papers were considered to have used this strategy
Provide interactive assistance			
13. Provide clinical supervision	24 (56%)	Provide clinicians with ongoing supervision focusing on the innovation. Provide training for clinical supervisors who will supervise clinicians who provide the innovation	Most papers using clinical supervision, integrated regular supervision into the intervention. Some papers studying a shorter clinical intervention period used clinical supervision only during a short implementation period. Spira *et al.* (2017) used clinical supervision during an initial implementation phase. Varghese *et al.* (2014) assessed India's Yashoda programme which includes supervision as an integrated part of the programme (NIPI, 2010)
Adapt and tailor to context			
15. Tailor strategies	15 (35%)	Tailor the implementation strategies to address barriers and leverage facilitators that were identified through earlier data collection	Tailor strategies was often used in RCTs or larger studies that reported making adaptations after earlier phases such as Hirschhorn and colleagues’ (2015) use of a pilot and two phases of adaptation prior to an RCT. Spector *et al.* (2012) adapted the WHO SCC Program to the local context and was also considered to have used this strategy
Develop stakeholder interrelationships			
20. Organize clinician implementation team meetings	14 (33%)	Develop and support teams of clinicians who are implementing the innovation and give them protected time to reflect on the implementation effort, share lessons learned and support one another’s learning	Use of clinical implementation team meetings ranged from limiting meetings to the early implementation phase to a regular and integrated part of the intervention. Jennings *et al.* (2015) used clinical implementation team meetings in the planning and very early implementation phases (Jennings *et al.*, 2010) whereas Kumar *et al.* (2008) described monthly meetings to discuss experiences, challenges and strategies
Train and educate stakeholders			
15. Conduct educational meetings	30 (70%)	Hold meetings targeted toward different stakeholder groups (e.g. providers, administrators, other organizational stakeholders, and community, patient/consumer and family stakeholders) to teach them about the clinical innovation	Conduct educational meetings was usually employed as a strategy to train health care providers or community health workers in an intervention. Waiswa *et al.* (2017) used joint and separate learning sessions with health facility and community members to introduce or review quality improvement techniques. Karim *et al.* (2013) conducted trainings with ‘model families’ who adopt healthy newborn care practices
Support clinicians			
50. Revise professional roles	21 (49%)	Shift and revise roles among professionals who provide care, and redesign job characteristics	Revise professional roles was mostly used in community-level interventions where a community health worker was integrated into the existing health system and responsibilities such as postnatal care were shifted to the community level. Callaghan-Koru *et al.* (2013) described the shifting of newborn care tasks to the Health Surveillance Assistants. A facility-level example of revising professional roles is Varghese and colleagues’ (2014) assessment of India's Yashoda programme where postnatal breastfeeding support was shifted to Yashodas
Engage consumers			
53. Intervene with patients/consumers to enhance uptake and adherence	15 (35%)	Develop strategies with patients to encourage and problem solve around adherence	While the four papers (Dasgupta *et al.*, 1997; Ojofeitimi *et al.*, 2000; Parekh *et al.*, 2004; Aryeetey and Antwi, 2013) evaluating the implementation of the BFHI did not describe details of the specific local implementation, they were considered to have used strategies described in BFHI documents. Steps 3 (inform women about benefits and management of breastfeeding) and 5 (show mothers how to breastfeed) were considered to be intervening with patients (WHO and UNICEF, 2009). Baqui *et al.* (2008) described community mobilisers who disseminate newborn care messages and encourage care seeking
Utilize financial strategies			
58. Access new funding	10 (23%)	Access new or existing money to facilitate the implementation	Most papers considered to have used the strategy access new funding were employing paid community workers to provide newborn home visits. The ASHA programme described by Sinha *et al.* (2014) included a paid monetary incentive for ASHA workers to make six postnatal visits. The hospital-based intervention described by Iyengar *et al.* (2014) included visiting facilitators who worked with staff to remedy gaps in equipment using discretionary funds as well as to involve district-level officers to facilitate purchase of high value items, recruit staff or facilitate trainings
Change infrastructure			
70. Change service sites	19 (44%)	Change the location of clinical service sites to increase access	Change service site was used in papers implementing interventions at the community-level using home-visits and thus changing the service site for postnatal care from facilities to the home. Darmstadt *et al.* (2010) described a community-based intervention where community health workers made four postnatal home visits to negotiate preventive care practices and assess newborns for illness

a Number and per cent of all included studies. Numbers differ slightly from which only includes studies for which effect sizes were calculated.

Some strategies were used consistently across papers. For example, ‘revise professional roles’ was used mostly in the community setting where a community health worker was integrated into the existing health system and responsibilities such as postnatal care were delivered in the community setting. Callaghan-Koru *et al.* (2013) described the shifting of newborn care tasks to the Health Surveillance Assistants (community health workers) in Malawi. A facility-level example of revising professional roles included Varghese and colleagues’ (2014) assessment of India's Yashoda programme where facility-based postnatal breastfeeding support was shifted to Yashodas (volunteer, facility-based health workers who support women and newborns). However, many other strategies were applied very differently across studies. Use of ‘organize clinician implementation team meetings’ ranged from organization of meetings only in the initial implementation phase to organization of regular meetings as an integrated part of the intervention. Jennings *et al.* (2015) described the use of job aids to improve facility-based postnatal counselling and care in rural Benin. In this intervention, implementation team meetings were limited to planning and very early implementation phases (Jennings *et al.*, 2010). In contrast, Kumar *et al.* (2008) described a community-based behaviour change intervention which used regular monthly meetings with newborn-care stakeholders and community volunteers to discuss experiences, challenges and strategies.

**Figure 2:**
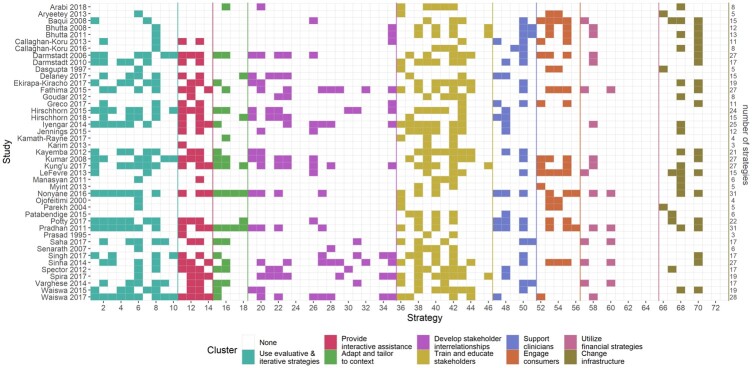
Use of implementation strategies. Strategy names and definitions in Supplementary file

A total of 14 strategies were not identified across any of the included papers; six of which belonged to one cluster: ‘utilize financial strategies’ (Figure 2).

### Description of implementation outcomes

**Figure 3:**
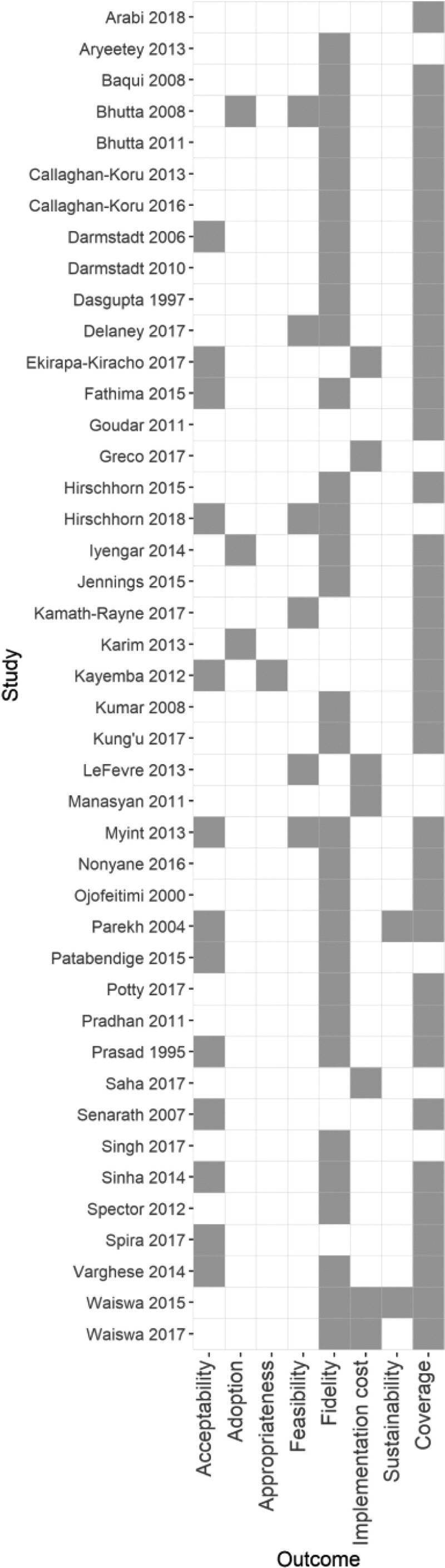
Implementation outcomes

The number of implementation outcomes reported ranged from one to four (inclusion criteria required at least one implementation outcome). Coverage and fidelity were the most frequently reported implementation outcomes, reported by 81% (*n* = 35) and 72% (*n* = 31) of papers, respectively (Figure 3). Other implementation outcomes as defined by Proctor *et al.* (2011) were infrequently reported. Acceptability was reported in 13 of the 43 papers, implementation cost in seven papers and feasibility in six papers.

**Figure 4:**
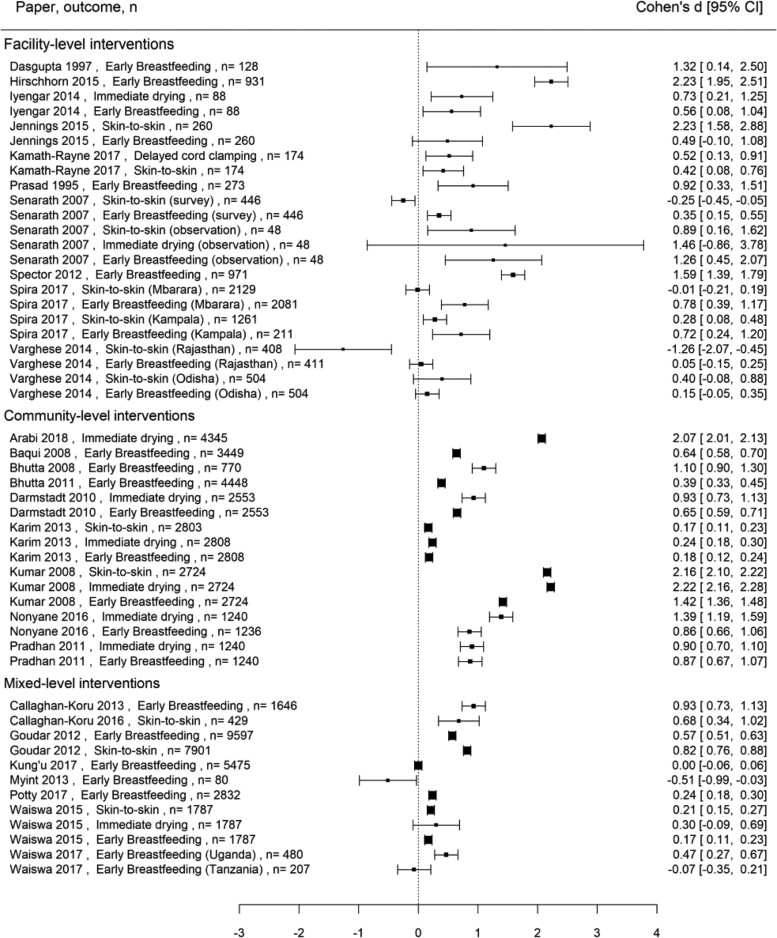
Forest plot of coverage outcomes

Coverage outcomes were presented either before and after the intervention or separately for an intervention and control group, with enough detail to calculate effect sizes for 51 outcomes in 27 papers. Coverage outcome effect sizes are presented in a forest plot in Figure 4. Standardized effect sizes (Cohen’s *d*) ranged from −1.26 to 2.23.

Fidelity in community-based studies was frequently reported as the number of community health worker visits received or mean time between birth and the first postnatal visit. For example, in a community-based skin-to-skin intervention evaluated by Darmstadt *et al.* (2006), it was intended for community health workers to visit women within 24 h of birth, and they reported a mean time of 7.8 h between birth and the first postnatal visit. The facility-based study using the SCC by Hirschhorn *et al.* (2015) reported fidelity to use of the checklist, where after coaching in the second adaptation of the intervention, the checklist was used at 88% of births.

Acceptability outcomes were reported in varying amounts of detail at both the client-level as well as the health care provider level. Varghese *et al.* (2014) reported detailed qualitative quotes from women and health care providers about the acceptability of the Yashoda programme. Nursing staff reported getting help from Yashodas and not needing to worry about mothers as the Yashodas would care for them. Women reported not wanting to stay in exclusive cabins as Yashodas did not cover these areas. Parekh *et al.* (2004) reported that only three women struggled with breastfeeding and all three were satisfied with advice given by healthcare providers.

Implementation cost was often reported in a specific economic evaluation, separate from a paper reporting the main results (coverage, clinical outcomes). Results were reported in many forms including annual cost, cost per live birth, cost per home visit, cost per disability adjusted life year (DALY) averted, and cost per life saved. Manasyan *et al.* (2011) reported differences in cost per DALY averted if equipment and training materials were reused. In this training for newborn care in urban first-level facilities, the total programme cost for 12 months (in 2015 US dollars) was $20 223.83 with a continuing cost of $14 128 per year. The programme led to a cost per life saved of $208 and cost per DALY averted of $5.24 which could be reduced to a cost per DALY averted of $1.84 if materials were reused. Ekirapa-Kiracho *et al.* (2017) reported a detailed analysis of costs, activities and time data, showing costs for difference phases of the project including design, set-up and implementation. Three scale-up scenarios were modelled and costs were compared with Uganda’s per capita public health expenditure, showing the additional cost of the programme was $1.04 per capita, representing 1.8% of the public health expenditure.

**Figure 5:**
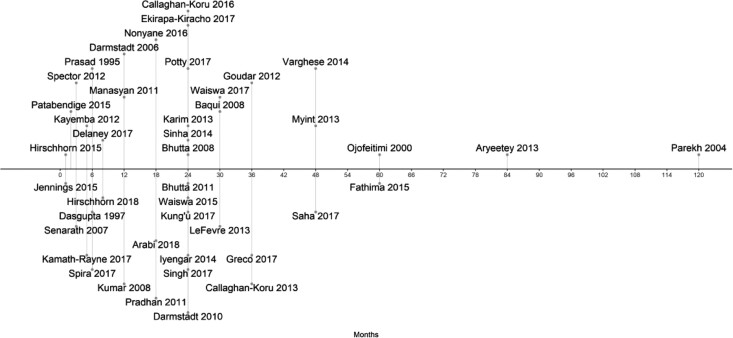
Timeline of interventions and evaluations

Sustainability was addressed specifically in only two included studies. For example, Parekh *et al.* (2004) evaluated progress in breastfeeding 10 years after the implementation of the BFHI. Figure 5 shows the elapsed time in months between the beginning of implementation and the beginning of evaluation. While several studies only evaluated the interventions within the same month or just a few months after implementation began, more than half of studies began evaluation 2 years after implementation began.

### Relationships between implementation strategies and coverage outcomes

**Figure 6:**
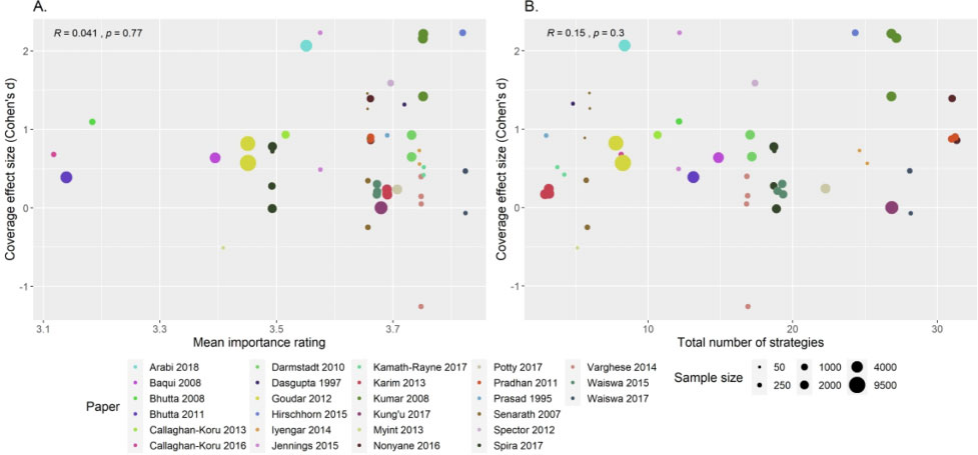
Importance ratings and effect sizes

For the 27 papers reporting a coverage outcome for which a standardized effect size could be calculated, Figure 6 shows scatter plots of the coverage effect size (Cohen’s *d*) and the mean rating of importance of strategies for each study or the total number of strategies used. Most studies used strategies with high importance ratings (>3.5), however, the full range of coverage effect sizes (−1.26 to 2.23) is seen at where importance ratings are high. The number of strategies used varied widely (3–31) and large effect sizes (*d* > 2) is seen at both the low (<10 strategies) and high (>25 strategies) ends of number of strategies used. We found no relationship between coverage and strategy importance ratings or number of strategies used (*r* = 0.4, *P* = 0.77 and *r* = 0.15, *P* = 0.3, respectively). In addition, we found no relationship between coverage effect size and proportion of strategies used within any of the nine implementation strategy clusters defined by Waltz *et al.* (2015) (scatter plots and correlation coefficients presented in the Supplementary file).

## Discussion

Interventions to save newborn lives are available but have not yet reached high coverage (Jones *et al.*, 2003; Darmstadt *et al.*, 2005; Bhutta *et al.*, 2013; Salam *et al.*, 2014). The most effective strategies with which to implement them, however, are not known. This is the first systematic review to examine implementation strategies used and outcomes reported in deploying ENC interventions. Key findings include that implementation efforts to integrate ENC interventions in low- and low middle-income countries have used a wide variety of implementation strategies but detailed reporting of the way strategies were applied and reporting of implementation outcomes beyond intervention coverage is limited. No specific strategy or cluster of strategies was associated with improved coverage. Understanding factors associated with successful implementation is crucial to improving coverage of interventions and sustaining them in practice.

We examined a wide range of implementation strategies but were unable to identify specific strategies associated with improved coverage of ENC. Similarly, a review of reviews examining organization interventions to improve in-patient care (not limited to lower income countries) reviewed five strategies for change and found none had consistent effects across studies (Wensing *et al.*, 2006). A study of uptake of hepatitis C treatment in US Veterans Health Administration (VHA) sites showed the number of strategies used as well as the importance ratings was associated with increased treatment uptake (Rogal *et al.*, 2017). However, in this review we failed to replicate this relationship: neither the number of strategies used nor the mean importance rating of applied strategies was associated with increased coverage.

Heterogeneity of the included studies (methodologies, clinical interventions and countries) may have contributed to the lack of relationship found between implementation strategies and coverage. Furthermore, it is possible that the low- and low middle-income country setting differs too much from the setting where the ratings were developed (VHA). In addition, while the review of uptake of hepatitis C treatment included 80 sites with varying levels of complexity (volume, risk level of patients, services, research funding, etc.), all sites were large VHA medical centres or satellite sites within an integrated health care system. Conversely, included papers spanned 18 countries across three continents. Interventions were implemented at various system levels including community-based interventions and facility-based interventions. Rogal *et al.* (2017) collected implementation strategy data directly from VHA sites through an electronic survey where the sites themselves reported which strategies they used [from the 73 defined by Powell *et al.* (2015)]. In the current review, the strategies had to be extracted from published papers and additional documentation where identified. As such, strategy use was researcher-defined and dependent on information reported. Some assumptions were made, e.g. a study of the BFHI was assumed to have used all strategies in the WHO and UNICEF (2009) description of BFHI although it could not be confirmed if each strategy was actually used in the particular setting.

A review of guideline implementation strategies to improve obstetric care in LMICs found audit and feedback to be frequently used while education interventions were only used in two of nine studies (Stokes *et al.*, 2016). A review of implementation strategies for maternal and child health care in LMICs found distribution of educational materials was widely used but ineffective when applied alone, while audit and feedback had small to moderate positive effects (Althabe *et al.*, 2008). The review of guideline implementation for obstetric care found that clinical audit implemented by management to be associated with lack of staff motivation to change (Stokes *et al.*, 2016). We found education-related interventions to be the most frequently used implementation strategies for ENC in this context while audit and feedback was used in fewer than one-quarter of studies.

Fourteen strategies were not identified in any included studies, including six strategies within the ‘utilize financial strategies’ cluster. It is possible that the strategies were not used or were not reported in published papers or other programme documentation. Financial strategies have either been absent from other reviews of implementation strategies (Stokes *et al.*, 2016; Imamura *et al.*, 2017) or were largely absent from the relevant literature (Althabe *et al.*, 2008).

Educational outreach programmes, used in 11 studies in this review, may be an effective approach to address inequities and ensure good coverage but can be costly (Althabe *et al.*, 2008). For example, Darmstadt *et al.* (2010), used long training durations and frequent refresher sessions, although did not report on implementation cost. Althabe *et al.* (2008) suggested train-the-trainer strategies might improve replicability and cost-effectiveness. We found eight studies had used this strategy, however, implementation cost and sustainability were rarely reported in the included ENC implementation literature.

Coverage and fidelity were the most frequently reported implementation outcomes in this review. Other implementation outcomes as defined by Proctor *et al.* (2011) were infrequently reported. The lack of implementation outcome reporting has been noted in the literature previously (Gaglio *et al.*, 2013). Without sufficient detail on implementation outcomes, even where studies may report high coverage, quality of care and acceptability (to both health care providers and health service beneficiaries) of interventions may be lacking. The main outcome addressed in this review was coverage of care, but quality of care and measurement of quality of care are also essential to improving service delivery and saving lives (Kinney *et al.*, 2010).

Including process evaluations within studies can improve how we understand factors which influence implementation. Data collection to inform such evaluations should commence early in a project (Limbani *et al.*, 2019). The UK Medical Research Council (MRC) guidance recommends these evaluations should systematically assess quality of implementation and fidelity to planned components to elucidate factors related to context which may be associated with variations in outcomes and could clarify causal mechanisms (Moore *et al.*, 2015). While the MRC guidance does not include a reporting checklist for process evaluations due to variability in methodology, Pinnock *et al.* (2017) developed standards for reporting on implementation studies (StaRI) which included guidance to enable researchers to describe implementation strategies used, alongside reporting of intervention effectiveness. Improving implementation research can improve service delivery and inform health policy design (Theobald *et al.*, 2018).

This is the first review to evaluate the use of implementation strategies as defined by Powell *et al.* (2012) and the strategy importance ratings established by Waltz *et al.* (2014) for ENC in low- and low middle-income countries. Strengths include a systematic literature search and review process. In addition, comprehensive frameworks of well-defined implementation strategies and implementation-outcomes were used. However, several limitations should be noted. Due to time and budget constraints, our literature search was limited to peer-reviewed and published literature which possibly excluded interventions described in the grey literature. While data on implementation strategies were extracted from the papers and linked documentation (protocols, programmatic documentation), additional strategies may have been used but not reported. Equally, strategies may have been reported in study protocols but not used in practice (or not well applied), biasing associations between strategies and effect sizes. In addition, use of strategies varied greatly between interventions from brief to fully integrated, rigorous use and this variability was not accounted for in quantitative analyses. Furthermore, as all associations explored in this review were observational, causality cannot be assumed.

Papers reporting on ENC interventions were excluded from the review either because they did not report any detail regarding implementation and/or did not report on implementation outcomes. These are not unique issues to the ENC literature and are widely recognized barriers in the implementation science literature (Michie *et al.*, 2009; McKibbon *et al.*, 2010; Proctor *et al.*, 2011). To overcome these barriers, further implementation and intervention information was sought from additional programme documentation of included papers where possible.

The framework of implementation strategies used in this review have an original basis in a compilation of strategies for use in health and mental health care (Powell *et al.*, 2012). In the absence of such a comprehensive list of defined implementation strategies specifically for newborn health or the low- and low middle-income country setting, the strategies defined by Powell *et al.* (2015) proved useful for describing strategies used in implementing ENC interventions. No implementation strategies were identified in included studies that did not fit into a pre-defined strategy from the framework. While the strategies themselves originated in the general health and mental health literature, the importance ratings used in the analysis were specifically established to facilitate the use of evidence-based programmes for VHA mental health services (Waltz *et al.*, 2014).

## Conclusions

This review highlighted several challenges in learning from implementation of ENC in low- and low middle-income countries, particularly poor description of interventions and reporting implementation outcomes. We were not able to show an association between implementation strategies and coverage of ENC although it has been shown in other contexts. There may be a number of reasons for this—including the quality and heterogeneity of the evidence considered in this review. Further research is needed to determine effectiveness of implementation strategies for improved coverage of newborn care in low-income settings. We recommend that policy makers and clinicians conducting research in newborn care in low-income settings report sufficient details on implementation strategies and outcomes and recommend use of the UK MRC guidance for process evaluations and the StaRI checklist for reporting on implementation studies. Improved reporting could enable the global newborn care community to learn from these experiences, with potential to improve service delivery and health policy as a result.

## Funding

Funding to support this study was from a graduate research scholarship from King’s College London, Centre for Doctoral Studies [Health Faculties US Scholarship]. This article is part of the supplement ‘nnovations in Implementation Research in Low- and Middle-Income Countries’, a collaboration of the Alliance for Health Policy and Systems Research and Health Policy and Planning. The supplement and this article were produced with financial support from the Alliance for Health Policy and Systems Research. The Alliance is able to conduct its work thanks to the commitment and support from a variety of funders. These include our long-term core contributors from national governments and international institutions, as well as designated funding for specific projects within our current priorities. For the full list of Alliance donors, please visit: https://www.who.int/alliance-hpsr/partners/en/.

## Author contributions

K.P. led and participated in all phases of the study including conceptualization, design, screening, data extraction, quality appraisal, synthesis, data analysis and interpretation. She also drafted the manuscript. C.T. participated in the conceptualization and design of the study, screening studies and interpretation of results. She revised and approved the final manuscript. E.P. participated in study screening, advised on data analysis and interpretation. He revised and approved the final manuscript. T.A.R. and J.H.N. participated in data extraction, quality appraisal and revised and approved the final manuscript. D.B. participated in the conceptualization and design of the study and interpretation of results. She revised and approved the final manuscript. All authors read and approved the final manuscript.

*Conflict of interest statement*. None declared.

*Ethical approval.* No ethical approval was required for this study.

## Supplementary Material

czaa122_Supplementary_File

## References

[czaa122-B1] Althabe F, Bergel E, Cafferata ML et al 2008. Strategies for improving the quality of health care in maternal and child health in low- and middle-income countries: an overview of systematic reviews. Paediatric and Perinatal Epidemiology 22: 42–60. 18237352 10.1111/j.1365-3016.2007.00912.x

[czaa122-B4103556] Arabi AME, Ibrahim SA, Manar A-R et al 2018. Perinatal outcomes following Helping Babies Breathe training and regular peer–peer skills practice among village midwives in Sudan. Archives of Disease in Childhood 103: 24–7. 28821501 10.1136/archdischild-2017-312809

[czaa122-B2] Aromataris E, Munn Z (eds). 2017. *Joanna Briggs Institute Reviewer’s Manual*. https://reviewersmanual.joannabriggs.org/.

[czaa122-B276243] Aryeetey RNO, Antwi C. 2013. Re-assessment of selected Baby-Friendly maternity facilities in Accra, Ghana. International Breastfeeding Journal 8. 10.1186/1746-4358-8-15 PMC3832220 24216173

[czaa122-B9002783] Baqui AH, El-Arifeen S, Darmstadt GL et al 2008. Effect of community-based newborn-care intervention package implemented through two service-delivery strategies in Sylhet district, Bangladesh: a cluster-randomised controlled trial. The Lancet 371: 1936–44. 10.1016/S0140-6736(08)60835-1 18539225

[czaa122-B462021] Bhutta Z, Memon ZA, Soofi S et al 2008. Implementing community-based perinatal care: results from a pilot study in rural Pakistan. Bulletin of the World Health Organization 2008: 452–9. 10.2471/BLT.07.045849 PMC2647462 18568274

[czaa122-B2701275] Bhutta ZA, Soofi S, Cousens S et al 2011. Improvement of perinatal and newborn care in rural Pakistan through community-based strategies: a cluster-randomised effectiveness trial. The Lancet 377: 403–12. 10.1016/S0140-6736(10)62274-X 21239052

[czaa122-B3] Bhutta ZA , Das JK, Rizvi A et al 2013. Evidence-based interventions for improvement of maternal and child nutrition: what can be done and at what cost? The Lancet 382: 452–77. 10.1016/S0140-6736(13)60996-4 23746776

[czaa122-B4] Bhutta ZA, Das JK, Bahl R et al 2014. Can available interventions end preventable deaths in mothers, newborn babies, and stillbirths, and at what cost? The Lancet 384: 347–70. 10.1016/S0140-6736(14)60792-3 24853604

[czaa122-B8294115] Callaghan-Koru JA, Estifanos AS, Sheferaw ED et al 2016. Practice of skin-to-skin contact, exclusive breastfeeding and other newborn care interventions in Ethiopia following promotion by facility and community health workers: results from a prospective outcome evaluation. Acta Paediatrica 105: e568–76. 27644765 10.1111/apa.13597

[czaa122-B5] Callaghan-Koru JA, Nonyane BAS, Guenther T *et al* et al 2013. Contribution of community-based newborn health promotion to reducing inequities in healthy newborn care practices and knowledge: evidence of improvement from a three-district pilot program in Malawi. BMC Public Health 13: 1052. 24199832 10.1186/1471-2458-13-1052 PMC3833651

[czaa122-B6] Damschroder LJ , Aron DC, Keith RE *et al*et al 2009. Fostering implementation of health services research findings into practice: a consolidated framework for advancing implementation science. Implement Science 4: 50. 10.1186/1748-5908-4-50 PMC2736161 19664226

[czaa122-B7] Darmstadt GL, Bhutta ZA, Cousens S et al 2005. Evidence-based, cost-effective interventions: how many newborn babies can we save? The Lancet 365: 977–88. 10.1016/S0140-6736(05)71088-6 15767001

[czaa122-B8] Darmstadt GL, Kumar V, Yadav R et al 2006. Introduction of community-based skin-to-skin care in rural Uttar Pradesh, India. Journal of Perinatology 26: 597–604. 16915302 10.1038/sj.jp.7211569

[czaa122-B9] Darmstadt GL, Choi Y, Arifeen SE et al 2010. Evaluation of a cluster-randomized controlled trial of a package of community-based maternal and newborn interventions in Mirzapur, Bangladesh Jose M. Belizan (ed). PLoS One 5: e9696. 20352087 10.1371/journal.pone.0009696 PMC2844410

[czaa122-B10] Darmstadt GL, Kinney MV, Chopra M et al 2014. Who has been caring for the baby? The Lancet 384: 174–88. 10.1016/S0140-6736(14)60458-X 24853603

[czaa122-B81584983] Dasgupta A, Bhattacharya S, Das M *et al*. 1997. Breast feeding practices in ateaching hospital of Calcutta before and after the adoption of BFHI (Baby Friendly Hospital Initiative). Journal of the Indian Medical Association 95: 169. 9420392

[czaa122-B90426970] Delaney MM, Maji P, Kalita T et al 2017. Improving Adherence to Essential Birth Practices Using the WHO Safe Childbirth Checklist With Peer Coaching: Experience From 60 Public Health Facilities in Uttar Pradesh, India. Global Health: Science and Practice 5: 217–31. 10.9745/GHSP-D-16-00410 PMC5487085 28655800

[czaa122-B11] Eccles MP, Armstrong D, Baker R et al 2009. An implementation research agenda. Implementation Science 4. http://implementationscience.biomedcentral.com/articles/10.1186/1748-5908-4-18, accessed 17 November 2017. 10.1186/1748-5908-4-18 PMC2671479 19351400

[czaa122-B12] Ekirapa-Kiracho E, Barger D, Mayora C et al 2017. Uganda Newborn Study (UNEST) trial: community-based maternal and newborn care economic analysis. Health Policy and Planning 32(Suppl 1): ii42–52. 10.1093/heapol/czw092 28981763

[czaa122-B13] Fathima FN, Raju M, Varadharajan KS et al 2015. Assessment of ‘accredited social health activists’—a national community health volunteer scheme in Karnataka State, India. Journal of Health, Population, and Nutrition 33: 137. PMC4438657 25995730

[czaa122-B14] Gaglio B, Shoup JA, Glasgow RE et al 2013. The RE-AIM framework: a systematic review of use over time. American Journal of Public Health 103: e38–46. 10.2105/AJPH.2013.301299 PMC3698732 23597377

[czaa122-B15] Ghaffar A, Langlois EV, Rasanathan K et al 2017. Strengthening health systems through embedded research. Bulletin of the World Health Organization 95: 87. 28250505 10.2471/BLT.16.189126 PMC5327943

[czaa122-B16] Glasziou P, Altman DG, Bossuyt P et al 2014. Reducing waste from incomplete or unusable reports of biomedical research. The Lancet 383: 267–76. 10.1016/S0140-6736(13)62228-X 24411647

[czaa122-B14184615] Goudar SS, Dhaded SM, Mcclure EM et al 2012. ENC training reduces perinatal mortality in Karnataka, India. The Journal of Maternal-Fetal & Neonatal Medicine: The Official Journal of the European Association of Perinatal Medicine, the Federation of Asia and Oceania Perinatal Societies, the International Society of Perinatal Obstetricians 25: 568–74. 10.3109/14767058.2011.584088 PMC13280768 21793707

[czaa122-B9369603] Greco G, Daviaud E, Owen H et al 2017. Malawi three district evaluation: Community-based maternal and newborn care economic analysis. Health Policy and Planning 32(Suppl 1): ii64–74. 10.1093/heapol/czw079 28981762

[czaa122-B17] Hirschhorn LR, Semrau K, Kodkany B et al 2015. Learning before leaping: integration of an adaptive study design process prior to initiation of BetterBirth, a large-scale randomized controlled trial in Uttar Pradesh, India. Implementation Science: IS 10: 117. 26271331 10.1186/s13012-015-0309-y PMC4536663

[czaa122-B0817174] Hirschhorn LR, Krasne M, Maisonneuve J et al 2018. Integration of the Opportunity-Ability-Motivation behavior change framework into a coaching-based WHO Safe Childbirth Checklist program in India. International Journal of Gynecology & Obstetrics 142: 321–8. 29862506 10.1002/ijgo.12542 PMC6099329

[czaa122-B18] Imamura M, Kanguru L, Penfold S et al 2017. A systematic review of implementation strategies to deliver guidelines on obstetric care practice in low- and middle-income countries. International Journal of Gynecology & Obstetrics 136: 19–28. 28099701 10.1002/ijgo.12005

[czaa122-B3927876] Iyengar K, Jain M, Thomas S et al 2014. Adherence to evidence based care practices for childbirth before and after a quality improvement intervention in health facilities of Rajasthan, India. BMC Pregnancy and Childbirth 14: 270. 25117856 10.1186/1471-2393-14-270 PMC4141099

[czaa122-B19] Jennings L, Yebadokpo AS, Affo J, Agbogbe M et al 2010. Antenatal counseling in maternal and newborn care: use of job aids to improve health worker performance and maternal understanding in Benin. BMC Pregnancy and Childbirth 10. http://bmcpregnancychildbirth.biomedcentral.com/articles/10.1186/1471-2393-10-75, accessed 19 January 2018. 10.1186/1471-2393-10-75 PMC3002891 21092183

[czaa122-B20] Jennings L, Yebadokpo A, Affo J, Agbogbe M et al 2015. Use of job aids to improve facility-based postnatal counseling and care in rural Benin. Maternal and Child Health Journal 19: 557–65. 24916207 10.1007/s10995-014-1537-5

[czaa122-B21] Jones G, Steketee RW, Black RE, Bhutta ZA, Morris SS et al 2003. How many child deaths can we prevent this year? The Lancet 362: 65–71. 10.1016/S0140-6736(03)13811-1 12853204

[czaa122-B20826519] Kamath-Rayne BD, Josyula S, Rule ARL, Vasquez JC. 2017. Improvements in the delivery of resuscitation and newborn care after Helping Babies Breathe training. Journal of Perinatology 37: 1153–60. 28726790 10.1038/jp.2017.110

[czaa122-B11950054] Karim AM, Admassu K, Schellenberg J et al 2013. Effect of Ethiopia’s Health Extension Program on Maternal and Newborn Health Care Practices in 101 Rural Districts: A Dose-Response Study. PLoS ONE 8: e65160. 23750240 10.1371/journal.pone.0065160 PMC3672192

[czaa122-B87891516] Kayemba CN, Sengendo H, Ssekitooleko J et al 2012. Introduction of Newborn Care within Integrated Community Case Management in Uganda. The American Journal of Tropical Medicine and Hygiene 87(Suppl 5): 46–53. 23136277 10.4269/ajtmh.2012.12-0133 PMC3748521

[czaa122-B22] Kinney MV, Kerber KJ, Black RE et al 2010. Sub-Saharan Africa’s mothers, newborns, and children: where and why do they die? PLoS Medicine 7. https://www.ncbi.nlm.nih.gov/pmc/articles/PMC2888581/, accessed 3 October 2019. 10.1371/journal.pmed.1000294 PMC2888581 20574524

[czaa122-B23] Kumar V, Mohanty S, Kumar A et al 2008. Effect of community-based behaviour change management on neonatal mortality in Shivgarh, Uttar Pradesh, India: a cluster-randomised controlled trial. Lancet (British Edition) 372: 1151–62. 10.1016/S0140-6736(08)61483-X 18926277

[czaa122-B0855661] Kung'u JK, Pendame R, Ndiaye MB et al 2018. Integrating nutrition into health systems at community level: Impact evaluation of the communitybased maternal and neonatal health and nutrition projects in Ethiopia, Kenya, and Senegal. *Maternal & Child Nutrition* 14(Suppl 1): e12577. 10.1111/mcn.12577 PMC6865938 29493902

[czaa122-B24] Lajeunesse MJ. 2017. *metagear: Comprehensive Research Synthesis Tools for Systematic Reviews and Meta-analysis*. https://CRAN.R-project.org/package=metagear, accessed 7 February 2020.

[czaa122-B677832829] Lefevre AE, Shillcutt SD, Waters HR et al 2013. Economic evaluation of neonatal care packages in a cluster-randomized controlled trial in Sylhet, Bangladesh. Bulletin of the World Health Organization 91: 736–45. 24115797 10.2471/BLT.12.117127 PMC3791651

[czaa122-B25] Limbani F, Goudge J, Joshi R et al . 2019. Process evaluation in the field: global learnings from seven implementation research hypertension projects in low- and middle-income countries. BMC Public Health 19: 953. 31340828 10.1186/s12889-019-7261-8 PMC6651979

[czaa122-B26] Manasyan A, Chomba E, McClure EM et al 2011. Cost-effectiveness of essential newborn care training in urban first-level facilities. Pediatrics 127: e1176–1181. 21502223 10.1542/peds.2010-2158 PMC3387868

[czaa122-B27] Martines J, Paul VK, Bhutta ZA et al 2005. Neonatal survival: a call for action. The Lancet 365: 1189–97. 10.1016/S0140-6736(05)71882-1 15794974

[czaa122-B28] McKibbon KA, Lokker C, Wilczynski NL et al 2010. A cross-sectional study of the number and frequency of terms used to refer to knowledge translation in a body of health literature in 2006: a Tower of Babel? Implementation Science: IS 5: 16. 21080976 10.1186/1748-5908-5-16 PMC2834600

[czaa122-B29] Michie S, Fixsen D, Grimshaw JM, Eccles MP et al 2009. Specifying and reporting complex behaviour change interventions: the need for a scientific method. Implementation Science: IS 4: 40. 19607700 10.1186/1748-5908-4-40 PMC2717906

[czaa122-B30] Moher D, Liberati A, Tetzlaff J, Altman DG, The PRISMA Group et al 2009. Preferred reporting items for systematic reviews and meta-analyses: the PRISMA statement. PLoS Medicine 6: e1000097. 19621072 10.1371/journal.pmed.1000097 PMC2707599

[czaa122-B31] Moore GF, Audrey S, Barker M et al 2015. Process evaluation of complex interventions: medical Research Council guidance. BMJ 350. https://www.bmj.com/content/350/bmj.h1258, accessed 20 February 2020. 10.1136/bmj.h1258 PMC4366184 25791983

[czaa122-B7495822] Myint T, Tint HS, Htet K et al 2013. Providers’ and clients’ perceptions and problems in providing newborn health services in project and non-project townships of Magway Region. Myanmar Health Sciences Research Journal 25: 106.

[czaa122-B32] National Health Mission. 2013. *About ASHA—Government of India*. http://nhm.gov.in/communitisation/asha/about-asha.html, accessed 27 December 2017.

[czaa122-B6712361] Nonyane BA, Kc A, Callaghan-Koru JA et al 2016. Equity improvements in maternal and newborn care indicators: results from the Bardiya district of Nepal. Health Policy and Planning 31: 405–14. 26303057 10.1093/heapol/czv077 PMC4986239

[czaa122-B7037479] Ojofeitimi EO, Esimai OA, Owolabi OO et al 2000. Breast Feeding Practices in Urban and Rural Health Centres: Impact of Baby Friendly Hospital Initiative in Ile-Ife, Nigeria. Nutrition and Health 14: 119–25. 10904936 10.1177/026010600001400204

[czaa122-B33] Parekh C, Bavdekar SB, Shaharao V et al 2004. Study of infant feeding practices: factors associated with faulty feeding. Journal of Tropical Pediatrics 50: 306–8. 15510764 10.1093/tropej/50.5.306

[czaa122-B2144639] Patabendige M, Senanayake H. 2015. Implementation of the WHO safe childbirth checklist program at a tertiary care setting in Sri Lanka: a developing country experience. BMC Pregnancy and Childbirth 15: 12. 25648543 10.1186/s12884-015-0436-0 PMC4324022

[czaa122-B34] Pinnock H, Barwick M, Carpenter CR et al 2017. Standards for reporting implementation studies (StaRI) statement. BMJ 356. https://www.bmj.com/content/356/bmj.i6795, accessed 20 February 2020. 10.1136/bmj.i6795 PMC5421438 28264797

[czaa122-B2116487] Potty R, Lakkappa M, Kar A et al 2017. Influence of integrated community- and facility-based interventions on select maternal and neonatal outcomes in Northern Karnataka, India: Lessons for implementation and measurement. Indian Journal of Public Health 61: 19. 28218158 10.4103/0019-557X.200256

[czaa122-B35] Powell BJ, McMillen JC, Proctor EK et al 2012. A compilation of strategies for implementing clinical innovations in health and mental health. Medical Care Research and Review 69: 123–57. 22203646 10.1177/1077558711430690 PMC3524416

[czaa122-B36] Powell BJ, Waltz TJ, Chinman MJ et al 2015. A refined compilation of implementation strategies: results from the Expert Recommendations for Implementing Change (ERIC) project. Implementation Science 10. http://implementationscience.biomedcentral.com/articles/10.1186/s13012-015-0209-1, accessed 17 November 2017. 10.1186/s13012-015-0209-1 PMC4328074 25889199

[czaa122-B7582447] Prasad B, Costello A. 1995. Impact and sustainability of a “baby friendly” health education intervention at a district hospital in Bihar, India. BMJ*:* British Medical Journal (International Edition) 310: 621–3. 7703747 10.1136/bmj.310.6980.621 PMC2549005

[czaa122-B3252377] Pradhan YV Upreti SR KC NP *et al*. 2011. Fitting Community Based Newborn Care Package into the health systems of Nepal. Journal of Nepal Health Research Council 9. 22929840

[czaa122-B37] Proctor E, Silmere H, Raghavan R et al 2011. Outcomes for Implementation research: conceptual distinctions, measurement challenges, and Research agenda. Administration and Policy in Mental Health 38: 65–76. 20957426 10.1007/s10488-010-0319-7 PMC3068522

[czaa122-B38] Proctor EK, Powell BJ, McMillen JC et al 2013. Implementation strategies: recommendations for specifying and reporting. Implementation Science 8: 139. 24289295 10.1186/1748-5908-8-139 PMC3882890

[czaa122-B39] R Core Team. 2018. R: A Language and Environment for Statistical Computing. Vienna, Austria: R Foundation for Statistical Computing. http://www.R-project.org/, accessed 10 July 2018.

[czaa122-B40] Rogal SS, Yakovchenko V, Waltz TJ et al 2017. The association between implementation strategy use and the uptake of hepatitis C treatment in a national sample. Implementation Science 12: 60. 28494811 10.1186/s13012-017-0588-6 PMC5425997

[czaa122-B41] Saha S , Varghese B. 2017. Cost-effectiveness of the Yashoda Programme: a facility-based mother and newborn support intervention in India. Journal of Health Management 19: 255–63.

[czaa122-B42] Salam RA, Mansoor T, Mallick D et al 2014. Essential childbirth and postnatal interventions for improved maternal and neonatal health. Reproductive Health 11(Suppl 1): S3. 10.1186/1742-4755-11-S1-S3 PMC4145857 25177795

[czaa122-B68955775] Senarath U, Fernando DN, Rodrigo I. 2007. Effect of Training for Care Providers on Practice of Essential Newborn Care in Hospitals in Sri Lanka. Journal of Obstetric, Gynecologic & Neonatal Nursing 36: 531–41. 10.1111/j.1552-6909.2007.00183.x 17973696

[czaa122-B7968081] Singh V, Ahmed S, Dreyfuss ML et al 2017. Non-governmental organization facilitation of a community-based nutrition and health program: Effect on program exposure and associated infant feeding practices in rural India. PLoS ONE 12: e0183316. 28910328 10.1371/journal.pone.0183316 PMC5598933

[czaa122-B43] Sinha LN, Kaur P, Gupta R et al 2014. Newborn care practices and home-based postnatal newborn care programme—Mewat, Haryana, India, 2013. Western Pacific Surveillance and Response Journal: WPSAR 5: 22–9. 25649098 10.5365/WPSAR.2014.5.1.006 PMC4310710

[czaa122-B44] Spector JM, Agrawal P, Kodkany B et al 2012. Improving quality of care for maternal and newborn health: prospective pilot study of the WHO Safe Childbirth Checklist program Philippa Middleton (ed). PLoS One 7: e35151. 22615733 10.1371/journal.pone.0035151 PMC3353951

[czaa122-B45] Spira C, Kwizera A, Jacob S et al 2017. Improving the quality of maternity services in Uganda through accelerated implementation of essential interventions by healthcare professional associations. International Journal of Gynecology & Obstetrics 139: 107–13. 28632951 10.1002/ijgo.12241 PMC5591067

[czaa122-B46] Stokes T, Shaw EJ, Camosso-Stefinovic J, Imamura M, Kanguru L, Hussein J et al 2016. Barriers and enablers to guideline implementation strategies to improve obstetric care practice in low- and middle-income countries: a systematic review of qualitative evidence. Implementation Science 11: 144. 27770807 10.1186/s13012-016-0508-1 PMC5075167

[czaa122-B47] Theobald S, Brandes N, Gyapong M et al 2018. Implementation research: new imperatives and opportunities in global health. The Lancet 392: 2214–28. 10.1016/S0140-6736(18)32205-0 30314860

[czaa122-B48] The World Bank. 2019. *World Bank Country and Lending Groups*. https://datahelpdesk.worldbank.org/knowledgebase/articles/906519-world-bank-country-and-lending-groups, accessed 28 September 2019.

[czaa122-B49] UNICEF, WHO, World Bank Group, United Nationset al 2019. *Levels & Trends in Child Mortality 2019*. New York: United Nations Children’s Fund.

[czaa122-B50] Varghese B, Roy R, Saha S, Roalkvam S et al 2014. Fostering maternal and newborn care in India the Yashoda way: does this improve maternal and newborn care practices during institutional delivery? Hamid Reza Baradaran (ed). PLoS One 9: e84145. 24454718 10.1371/journal.pone.0084145 PMC3893122

[czaa122-B4868664] Waiswa P, Pariyo G, Kallander K et al 2015. Effect of the Uganda Newborn Study on care-seeking and care practices: a cluster-randomised controlled trial. Global Health Action 8: 24584. 25843498 10.3402/gha.v8.24584 PMC4385212

[czaa122-B51] Waiswa P, Manzi F, Mbaruku G et al 2017. Effects of the EQUIP quasi-experimental study testing a collaborative quality improvement approach for maternal and newborn health care in Tanzania and Uganda. Arafumin P, Akuze J, Baker U, Balidawa H, Jaribu J, Kajjo D, Kalungi J, Kawala B, Majura A, Mandu R, Msonde I, Okuga M, Saulnier D, Sedekia Y, Tancred T, Temu S (eds). Implementation Science: IS 12: 89. 10.1186/s13012-017-0604-x PMC5516352 28720114

[czaa122-B52] Waltz TJ, Powell BJ, Chinman MJ et al 2014. Expert Recommendations for Implementing Change (ERIC): protocol for a mixed methods study. Implementation Science 9: 39. 24669765 10.1186/1748-5908-9-39 PMC3987065

[czaa122-B53] Waltz TJ, Powell BJ, Matthieu MM et al 2015. Use of concept mapping to characterize relationships among implementation strategies and assess their feasibility and importance: results from the Expert Recommendations for Implementing Change (ERIC) study. Implementation Science 10. http://implementationscience.biomedcentral.com/articles/10.1186/s13012-015-0295-0, accessed 17 November 2017. 10.1186/s13012-015-0295-0 PMC4527340 26249843

[czaa122-B54] Wensing M, Wollersheim H, Grol R et al 2006. Organizational interventions to implement improvements in patient care: a structured review of reviews. Implementation Science 1:2. 16722567 10.1186/1748-5908-1-2 PMC1436010

[czaa122-B55] WHO. 2013. WHO Recommendations on Postnatal Care of the Mother and Newborn. 24624481

[czaa122-B56] WHO. 2016. *Investing in Knowledge for Resilient Health Systems: Strategic Plan 2016–2020*.

[czaa122-B57] WHO and UNICEF 2009. *Baby-friendly Hospital Initiative: Revised, Updated and Expanded for Integrated Care*. http://www.ncbi.nlm.nih.gov/books/NBK153471/, accessed 19 January 2018. 23926623

[czaa122-B58] World Health Organization. 2017. *WHO Recommendations on Newborn Health*. http://www.who.int/maternal_child_adolescent/documents/newborn-health-recommendations/en/, accessed 7 February 2020.

